# Application of artificial intelligence-assisted image diagnosis software based on volume data reconstruction technique in medical imaging practice teaching

**DOI:** 10.1186/s12909-024-05382-6

**Published:** 2024-04-11

**Authors:** DongXu Wang, BingCheng Huai, Xing Ma, BaiMing Jin, YuGuang Wang, MengYu Chen, JunZhi Sang, RuiNan Liu

**Affiliations:** 1 Department of Medical Imaging, Second Affiliated Hospital of Qiqihar Medical University, 37 West Zhonghua Road, Qiqihar, Heilongjiang 161006 China; 2 Center for Higher Education Research and Teaching Quality Evaluation, Harbin Medical University, Harbin, Heilongjiang 150000 China; 3 School of Public Health, Qiqihar Medical University, 333 BuKui North Street, Qiqihar, Heilongjiang 161006 China; 4 Academic Affairs Section, Second Affiliated Hospital of Qiqihar Medical University, 37 West Zhonghua Road, Qiqihar, Heilongjiang 161006 China

## Abstract

**Background:**

In medical imaging courses, due to the complexity of anatomical relationships, limited number of practical course hours and instructors, how to improve the teaching quality of practical skills and self-directed learning ability has always been a challenge for higher medical education. Artificial intelligence-assisted diagnostic (AISD) software based on volume data reconstruction (VDR) technique is gradually entering radiology. It converts two-dimensional images into three-dimensional images, and AI can assist in image diagnosis. However, the application of artificial intelligence in medical education is still in its early stages. The purpose of this study is to explore the application value of AISD software based on VDR technique in medical imaging practical teaching, and to provide a basis for improving medical imaging practical teaching.

**Methods:**

Totally 41 students majoring in clinical medicine in 2017 were enrolled as the experiment group. AISD software based on VDR was used in practical teaching of medical imaging to display 3D images and mark lesions with AISD. Then annotations were provided and diagnostic suggestions were given. Also 43 students majoring in clinical medicine from 2016 were chosen as the control group, who were taught with the conventional film and multimedia teaching methods. The exam results and evaluation scales were compared statistically between groups.

**Results:**

The total skill scores of the test group were significantly higher compared with the control group (84.51 ± 3.81 vs. 80.67 ± 5.43). The scores of computed tomography (CT) diagnosis (49.93 ± 3.59 vs. 46.60 ± 4.89) and magnetic resonance (MR) diagnosis (17.41 ± 1.00 vs. 16.93 ± 1.14) of the experiment group were both significantly higher. The scores of academic self-efficacy (82.17 ± 4.67) and self-directed learning ability (235.56 ± 13.50) of the group were significantly higher compared with the control group (78.93 ± 6.29, 226.35 ± 13.90).

**Conclusions:**

Applying AISD software based on VDR to medical imaging practice teaching can enable students to timely obtain AI annotated lesion information and 3D images, which may help improve their image reading skills and enhance their academic self-efficacy and self-directed learning abilities.

**Supplementary Information:**

The online version contains supplementary material available at 10.1186/s12909-024-05382-6.

## Introduction

Medical imaging is an important clinical medicine course with strong clinical practicality. Students need to master the imaging characteristics of normal imaging manifestations and common diseases [1, 2]. Due to the fact that medical images are two-dimensional cross-sectional images [3, 4], it is difficult for students to understand adjacent structures, which has become a difficulty in practical teaching of images. To understand the anatomic relationships on images and diagnostic imaging, students have to establish the three-dimensional (3D) images of diseases [5]. However, the traditional multimedium practical curriculum has four limitations. First, despite the use of PPT for explanation of imaging characteristics, not all typical imaging features can be observed during actual image reading, due to the limitation by the number of image films. Second, due to the impact of low film quality, the films will yellow after long time and show scratches. Moreover, the films are very small and cannot be well differentiated by beginners of undergraduate students. Third, the 3D space can be hardly established, and the students have no clear clues, so the students will not be interested in and are even unwilling to do practical learning. Fourth, due to the limitation of faculty strength and schooltime, not every case can be guided in real time to each student. Thus, it is important to allow students to establish 3D images in real time and learn efficiently through clinical cases during practical curriculum.

Medical imaging has undergone several transformations. How to improve the quality of practical skills teaching and self-directed learning ability of students has long been a challenge for higher medical education. Few universities have always adhered to traditional teaching practices. Considering the complexity of anatomical relationships in medical imaging courses, the limited duration of practical courses, and the number of instructors [6], we have chosen to apply artificial intelligence imaging assisted diagnosis (AISD) software based on volume data reconstruction (VDR) technique to the practical teaching of this study.

Along with the rapid development of the computer technology [7], the application of artificial intelligence (AI) in the medical field has become diversified [8], especially in AIassisted image diagnosis [9]. Artificial intelligence-assisted image diagnosis (AISD) system software is based on convolutional neural network as the framework to build a lesion detection model, and constantly increase the amount of data through twisting, cutting and other methods [10, 11]. Medical images are analyzed and abnormal areas are automatically identified and labeled to assist doctors in making clinical diagnoses. The application of AI in medicine will change radiology practices in various ways. People are increasingly realizing that medical education should now include the application of AI [12]. Applying AI to education has many benefits, including a large amount of data storage available for students to access, less teacher involvement, and the display of 3D images. As one of the important technologies in AISD software, volume data reconstruction (VDR) refers to the method of obtaining original data by scanning human organs, using computer post-processing and manual editing to synthesize three-dimensional images from multiple plane images. According to the corresponding principle, each type of voxel number value is processed, and finally the 3D stereoscopic images is displayed through different grayscales or colors. This technology is mainly applied to the display of bones, pulmonary nodules, blood vessels, etc., and the user can observe their structure in a three-dimensional, intuitive, and clear manner. At present, the application of AISD software based on VDR in medical education has not been fully explored. This study utilized AISD software to enhance students’ realistic experience, especially in complex anatomical areas of the human body.

On basis of VDR and image recognition technology, the artificial intelligence (AI) technology has also progressed extensively. We use AISD software based on VDR technique to reconstruct two-dimensional images into three-dimensional images to assist in rapid and accurate clinical diagnosis and treatment, while also bringing new opportunities and challenges to clinical imaging teaching. In this study, AISD software based on VDR was compared with traditional teaching, expecting to provide a basis for the improvement of radiology practice teaching.

This study has two objectives. The first objective is to apply the AISD software used by doctors to practical teaching of imaging, helping students observe images from a three-dimensional perspective and improving the quality of teaching practical skills. The second goal is to identify ways to enhance academic self-efficacy and self-directed learning abilities through the interaction between students and AISD software.

## Materials and methods

### AISD software

AISD software uses a cascaded Residual Network (ResU-net) algorithm to segment aneurysms on the original axial images in head and neck CT angiography, including ResU-net1 and ResU-net2 modules. The ResU-net1 network is used for aneurysm detection, with its encoder using 5-layer down-sampling and the decoder using 5-layer up-sampling. Input channel number is 1, input original axis image, output channel number is 2. If the size of the output result is the same as the input size, the number of channels becomes 2. Each voxel point in channel 1 represents the probability of an aneurysm. When the probability value is greater than 0.5, it is considered that the current voxel point is an aneurysm, which serves as the standard for AI diagnosis of aneurysms. Each voxel point in channel 2 represents the length, width, and height information of the aneurysm when the current voxel point is the center point of the aneurysm. It will cut the detected aneurysm into 48 × 48 × 48 pixel data cubes and input them into the ResU-net2 network for aneurysm segmentation. The ResU-net2 network encoder uses 3 layers of down-sampling, while the decoder uses 3 layers of up-sampling. After outputting the segmentation result of the aneurysm, the generated aneurysm segmentation result is restored to the corresponding position in the image through post-processing. Using Digital Subtraction Angiography as the gold standard, the sensitivity, specificity, and accuracy of diagnosing intracranial aneurysms were 95.9%, 92.4%, and 95.4%, respectively. The automatic detection of fractures is based on V-Net. V-Net provides a 3D convolutional neural network architecture for extracting fracture features and locating images. The neural network first extracts corresponding features through the image feature compression network path, and then recovers to a three-dimensional matrix of the same size as the input through a decompressing network symmetric to the compression network. The software processing result interface includes horizontal axis images with rib fracture markers, curved planar reconstruction images, and VDR images to facilitate diagnostic physicians in observing the fracture situation detected by AI. The software processing result page includes horizontal axis images with rib positioning labels and fracture site markers. Users can simultaneously perform post-processing of Multi Planar Reconstruction (MPR) and VDR in the software to observe rib fractures detected by AI from multiple perspectives. When diagnosing rib fractures, the software can also automatically represent the type of fracture, such as displaced fractures, non-displaced fractures, and old fractures. Post-processing technology of MPR and 3D is used to help identify fractures and determine the nature of fractures. Its detection rates for dislocation fractures, non-dislocation fractures, and old fractures are 96.04%, 97.02%, and 98.79%, respectively. The total missed diagnosis rate for different types of rib fractures is 2.53%. Pulmonary nodule recognition utilizes U-net networks for lung segmentation. The networks separate the lung area from the background and other organs, retaining only CT images containing lung information. This program utilizes Retina-Net network for lung tissue nodule detection, locates all possible candidate regions of lung nodules in lung CT images, and outputs their positions and confidence levels. It then uses the Non-Maximum Suppression algorithm to screen candidate regions for pulmonary nodules, removing duplicate or low confidence candidate regions and preserving the final pulmonary nodule detection results. After cropping and scaling each candidate nodule, the program obtains a uniformly sized small image block as input to the segmentation network. It sets the size of small image blocks to 64 × 64 to ensure consistent input size for the segmentation network, while avoiding the impact of excessively large or small image blocks on the segmentation results. DeepLabv3 + network is used for lung nodule segmentation, which separates the lung nodules in small images from the background and outputs the segmentation results. At the same time, conditional random field algorithm is used to optimize and refine the lung nodule segmentation results. Dense-Net network is used for lung nodule classification, which distinguishes lung nodules in small image blocks from the background and outputs classification results. The cross-entropy damage function and accuracy are used as optimization objectives and evaluation indicators for the classification model, respectively, to measure the prediction error and accuracy of the model for the category of pulmonary nodules. The detection method for pulmonary nodules has sensitivity, specificity, and accuracy indicators of 95%, 98%, and 96%, respectively.

### Design

**Fig. 1:**
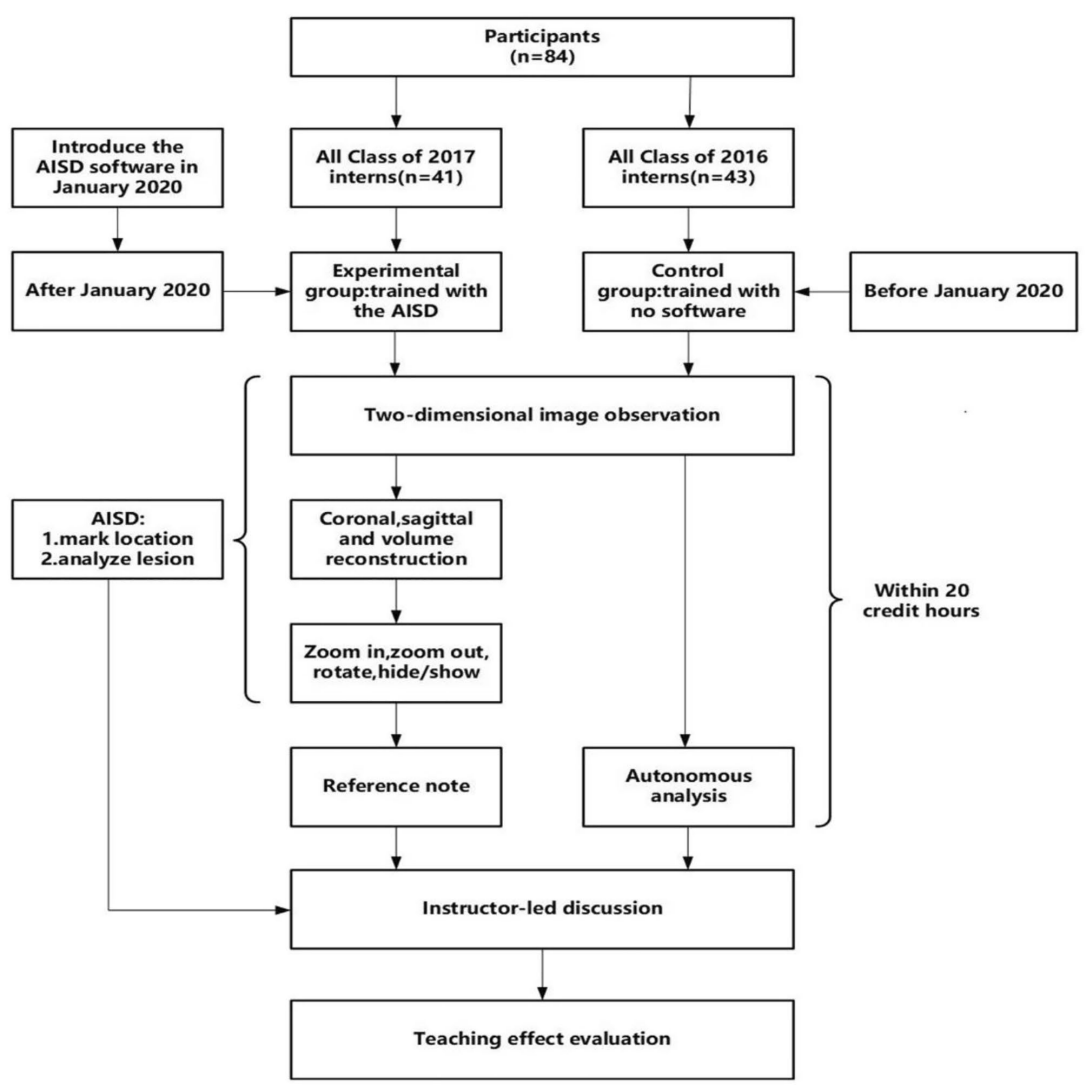
Research process and teaching implementation flowchart

The AISD software based on VDR (Shukun Internet Sci & Tec Co., Ltd., Beijing) was installed in January 2020 and used in the practical teaching of the 2017 grade students, while the previous students used traditional teaching (video: AISD operation video and function demonstration). The data in the form of Digital Imaging and Communications in Medicine (DICOM) from the Picture Archiving and Communication System (PACS) of our hospital was imported to the software. The 3D images were displayed by endowing the tissues with colors via the software. Meanwhile, trans axial, coronal and sagittal images were displayed. The students can magnify, minify, revolve, adjust windows, and hide/display images. The lesions were marked, and the students’ diagnostic suggestions were given. Then the images were analyzed with AI and the lesions were re-marked, which were pushed to the students (Fig. 1). The two groups were both taught by the same imaging teacher who had 10 years of clinical experience. All procedures performed in studies involving human participants were in accordance with the ethical standards of the institutional and/or national research committee and with the 1964 Helsinki declaration and its later amendments or comparable ethical standards. This study was approved by the Ethics Committee of the Second Affiliated Hospital of Qiqihar Medical College (No. 20,190,017) and obtained the written informed consent of all participants.

#### Participants

In January 2020, AISD software based on VDR technique (Shukun Network Technology Co., Ltd., Beijing) was installed and applied in practical teaching of image reading. The student group was based on this time point as the boundary. Prior to this date, all 2016 students who came to the hospital for internships did not use AISD software and were designated as the control group. After this date, all students who came to the hospital for internships in 2017 would receive AISD software learning and be designated as the experiment group. This comparative study using previous and subsequent students avoided psychological imbalance between two groups of students and bias in the final data results. Totally 41 students majoring in clinical medicine in 2017 from Qiqihar Medical University were enrolled as the experiment group, including 18 males and 23 females and aged 19–25 years old (21.73 ± 1.41 years). The comprehensive measuring and evaluating scores at 1 year before the enrollment were 84.73 ± 11.72. Then 43 students majoring in clinical medicine in 2016 were enrolled as the control group, including 19 males, 24 females and aged 20–27 years old (22.35 ± 1.65 years). The comprehensive measuring and evaluating scores at 1 year before the enrollment were 83.98 ± 9.58. No significant difference was found in the baseline data between the two groups (*P* > 0.05). The practical course of teaching was Medical Imaging with 20 classes. The contents, hours and textbook of teaching were all the same between the two groups. The teachers were totally the same between the two groups.

### Experimental procedure

**Fig. 2:**
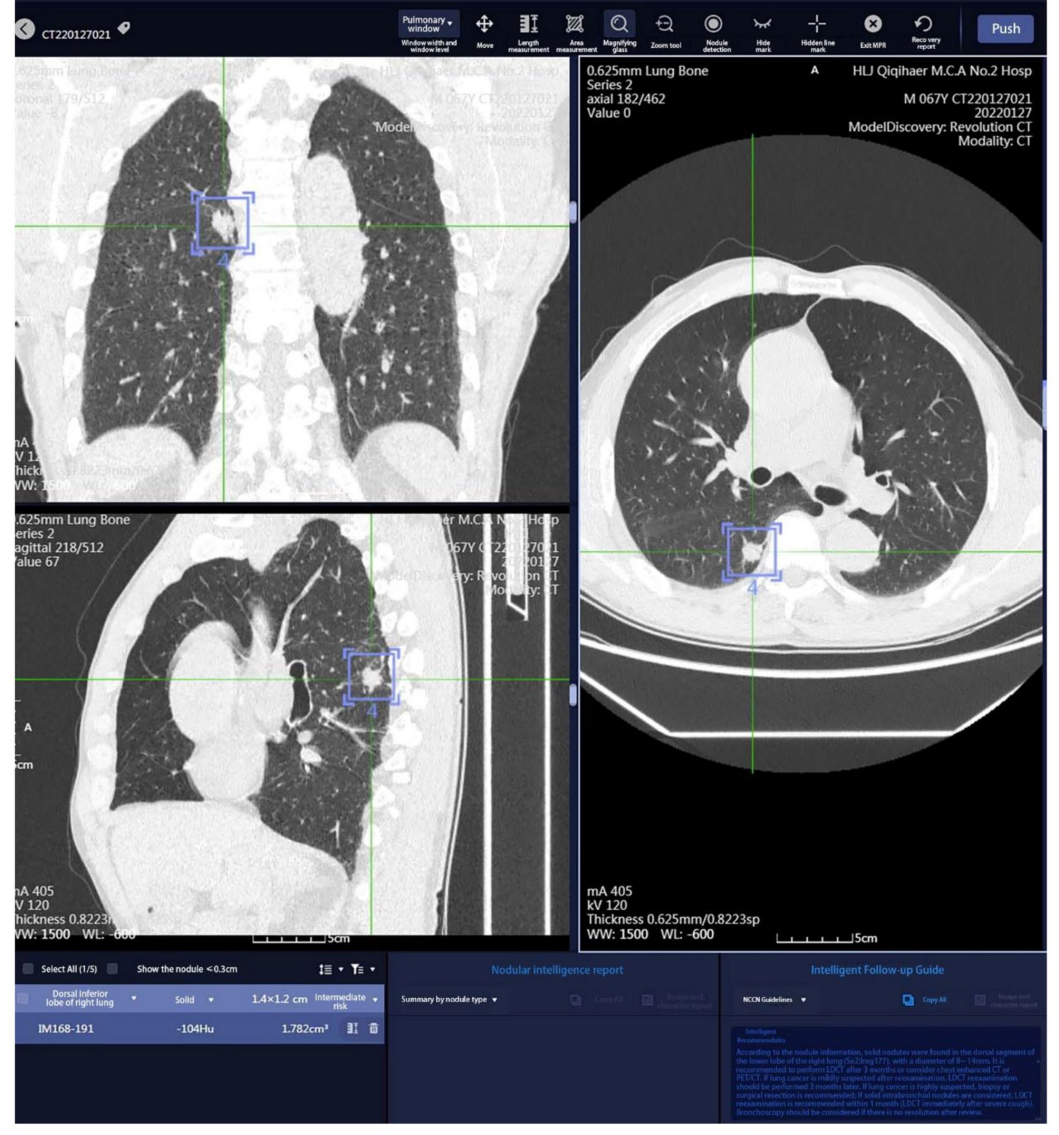
Software images of respiratory cases the software can display transaxial and sagittal images. The AI circles marked the position (dorsal inferior lobe of right lung), size (1.4 cm×1.2 cm), volume (1.782 cm^3^) and density (-104 Hu) of the lesions, and presented the suggestions on the follow-up to the lesions

**Fig. 3:**
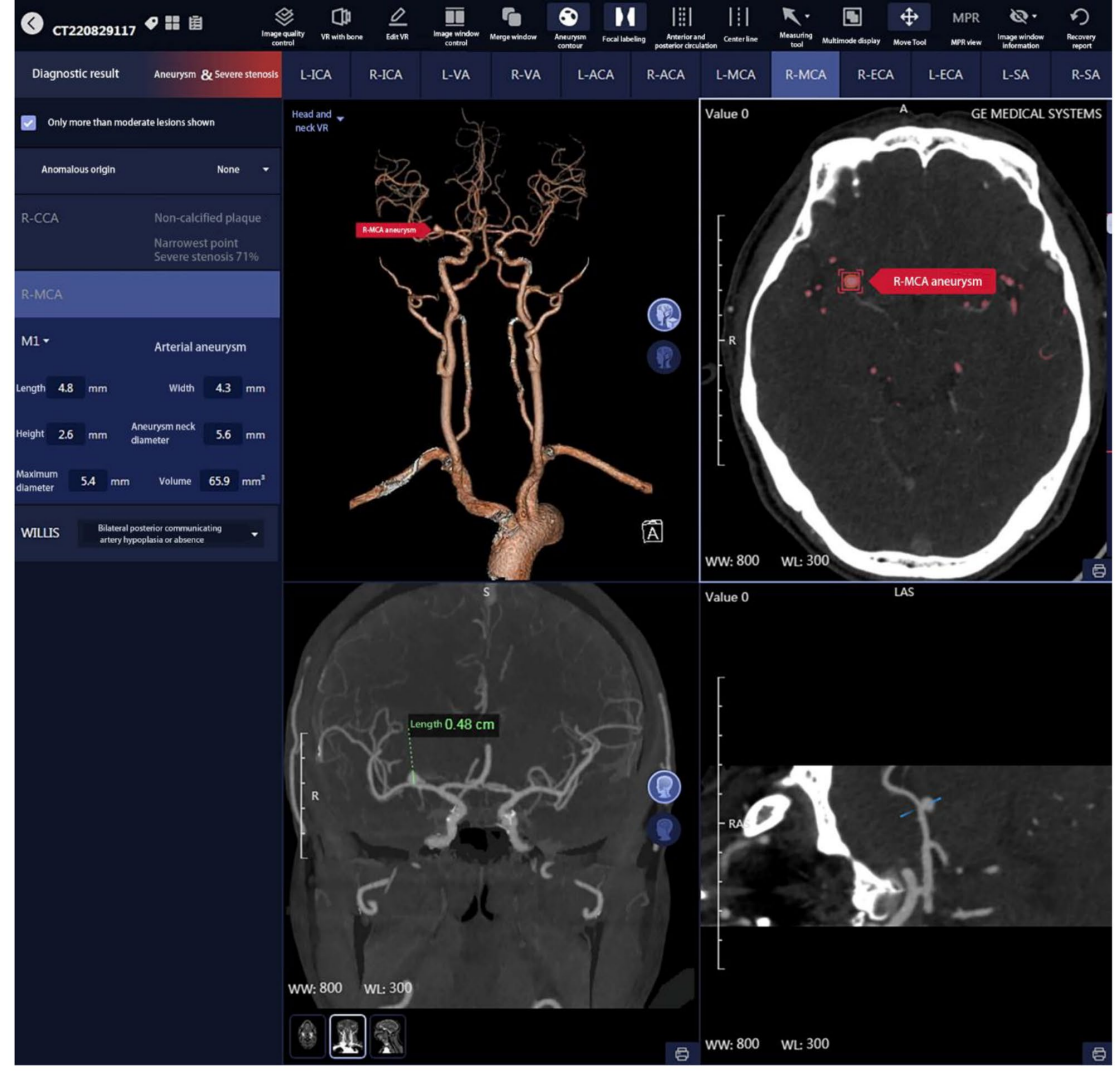
Software images of head and neck vascular cases. The software provides VDR stereoscopic images (position of aneurysm marked by AI) that can be revolved and magnified, and offers horizontal images (positions of lesions marked on AI circles), coronal images (AI measured aneurysm size = 4.8 mm×4.3 mm×2.6 mm, volume = 65.9 mm^3^), and stretched camber images of blood vessels at the lesions. It also displays a complete relationship between blood vessels and lesions on the plane view

**Fig. 4:**
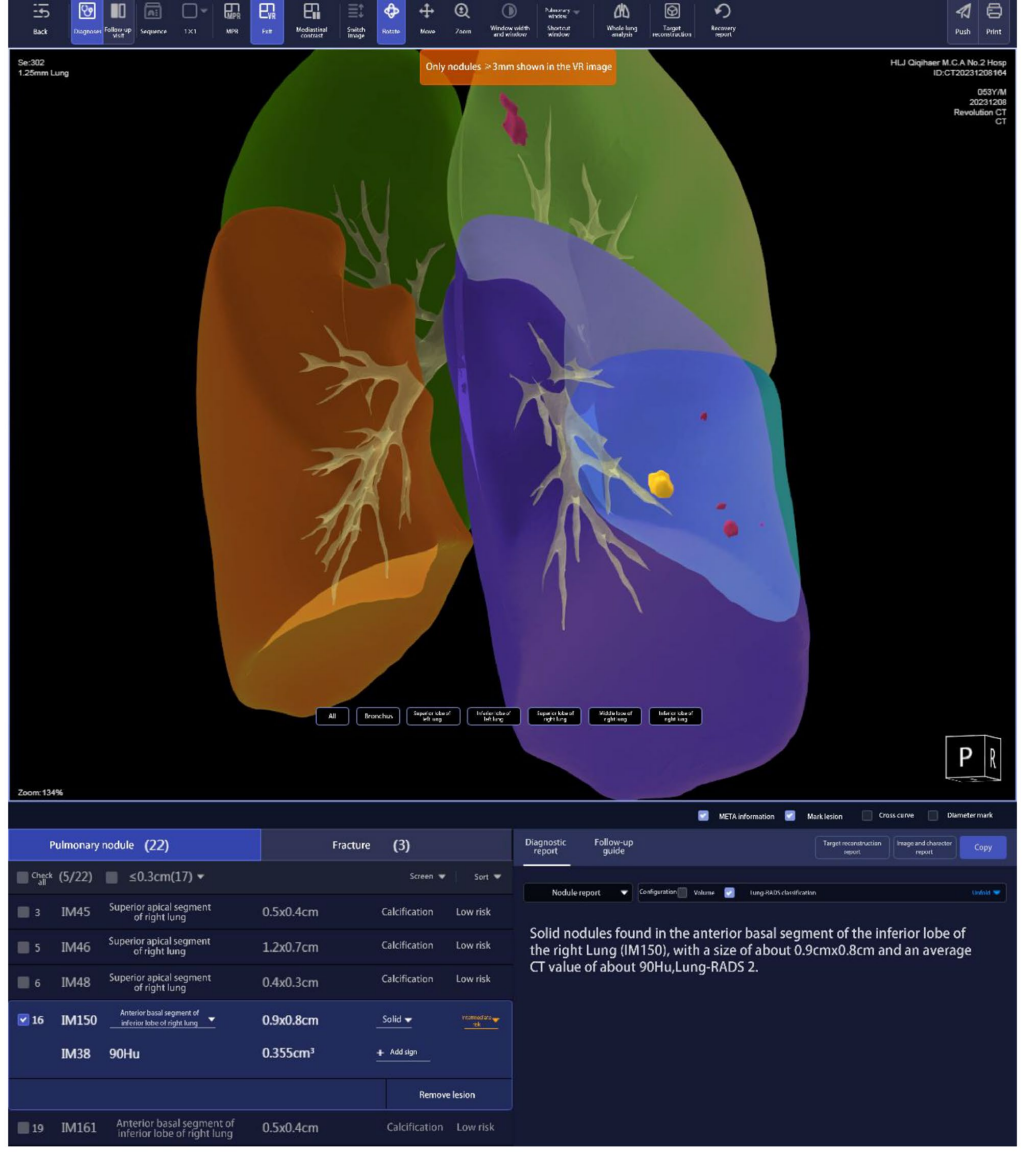
VDR screenshot of respiratory system case. In three-dimensional images, lesions were displayed in the form of pink and yellow nodules, and the distribution and surface morphology of lesions in the lobes and segments of the lungs, as well as the relationship between surrounding pulmonary blood vessels and bronchi, were clearly displayed

Implementation of teaching in the experiment group: The medical history, physical examination, and imaging of typical clinical cases were collected as per the syllabus. The image data of computerized tomography (CT), magnetic resonance (MR) and enhanced scan from the PACS were imported to the software. Then transaxial, coronal and sagittal images were generated, which clearly showed the relationship between lesions and the peripheral anatomic structures. The positions, sizes and properties of the lesions were analyzed via AI (Fig. 2). Before the teaching, the students were informed about the concrete cases. During the explanation, the students were guided stepwise to analyze the cases. Then questions were raised for the students to discuss, and finally the teacher summarized the lessons. During the teaching, the images of the cases were sent to coronal and sagittal reconstruction and VDR on the software, so the students can revolve and observe the sizes, extents, and relationships with adjacent blood vessels from multiple perspectives. After the students marked the lesions, they can submit it to the software. Then after image analysis via AI, text annotations were generated (Fig. 3), which were pushed again to the students. Each student can search the cases of interest in the software for learning. Each case was annotated with diagnostic imaging characteristics via AI, which helped with learning. Taking respiratory system practice image reading as an example, AISD software automatically identifies suspicious lesions in the entire lung of selected cases, and can label and report the location, size, morphology, nodule type, CT value, lung imaging reporting and data system score, likelihood of nodule malignancy, etc. Through AI diagnosis results, detailed information of corresponding lesions can be viewed. At the same time, by using the mouse and keyboard to zoom in, out, and rotate the VDR stereoscopic image, the surface morphology of the lesion nodule and its relationship with surrounding pulmonary blood vessels and bronchi can be observed more clearly (Fig. 4), and the subtle changes in its own structure and surrounding areas can be more fully displayed.

Implementation of teaching to the control group: The traditional teaching method was used in the control group. Before the teaching, the students were informed about the concrete cases, and the cases were explained to them via PPT. During the explanation combine the students were directed stepwise to analyze the cases. Images were played via PPT, and real image films were presented to the students. Then questions were raised for the students to discuss, and finally the teacher summarized the lessons. According to the syllabus, the teacher chose typical images from the PACS, and made PPT of the cases. During the teaching process, students analyzed and learned abnormal imaging signs through continuous observation at the two-dimensional image level in PACS, and difficult images were assisted by teachers to answer.

### Evaluation of teaching effect

#### Final exam

Theoretical knowledge exam and image reading skill exam were adopted. The total score of theoretical exams was 100, including 20 for term explanation, 30 for brief questions, 10 for discussion, and 40 for single choices. In the image reading skill exam, 50 reading images for single choices of diagnosis were made as per the syllabus, which included X-ray diagnosis [20], CT diagnosis (60), and MR diagnosis [20]. The time of the exam was 60 min. All images were real cases chosen from PACS.

#### Academic self-efficacy scale

This scale was compiled by Liang Yusong from Central China Normal University [13]. The academic self-efficacy was divided into two independent dimensions, including learning ability self-efficacy, and learning behavior self-efficacy [7]. The learning ability self-efficacy (11 terms) refers to the evaluation whether a student can accomplish the academic affair, achieve satisfactory result, and avoid failure. The learning behavior self-efficacy (11 terms) refers to the evaluation whether a student thinks his/her learning behavior can achieve the learning objective. Each term has 5 grades (1–5 scores): fully unqualified (1 score), very unqualified (2 scores), a bit qualified (3 scores), very qualified (4 scores) and fully qualified (5 scores).

#### Self-directed learning scale

This scale involves 5 dimensions: consciousness of learning, learning behavior, learning strategy, learning evaluation, and interpersonal skills, and each dimension has 12 terms. Each term has 5 grades (1–5 scores): never (1 score), seldom (2 scores), occasionally (3 scores), often (4 scores) and always (5 scores). Each student chose the most appropriate answer according to his/her thoughts and feelings about learning. A larger score means the student has stronger self-directed learning ability.

### Statistical analysis

Data were analyzed on SPSS 18.0. Shapiro Wilk test for normal distribution test was adopted. The Quantitative data were expressed as Mean ± Standard Deviation (SD), and Shapiro-Wilk test was used for normal distribution test. When the data were normally distributed and showed equal variance within groups, student t test was performed; otherwise, Mann-Whitney U test was used. Qualitative data are expressed as percentages and Chi-square tests were used for comparison. *P* < 0.05 was considered to be statistically significant.

## Results

**Fig. 5:**
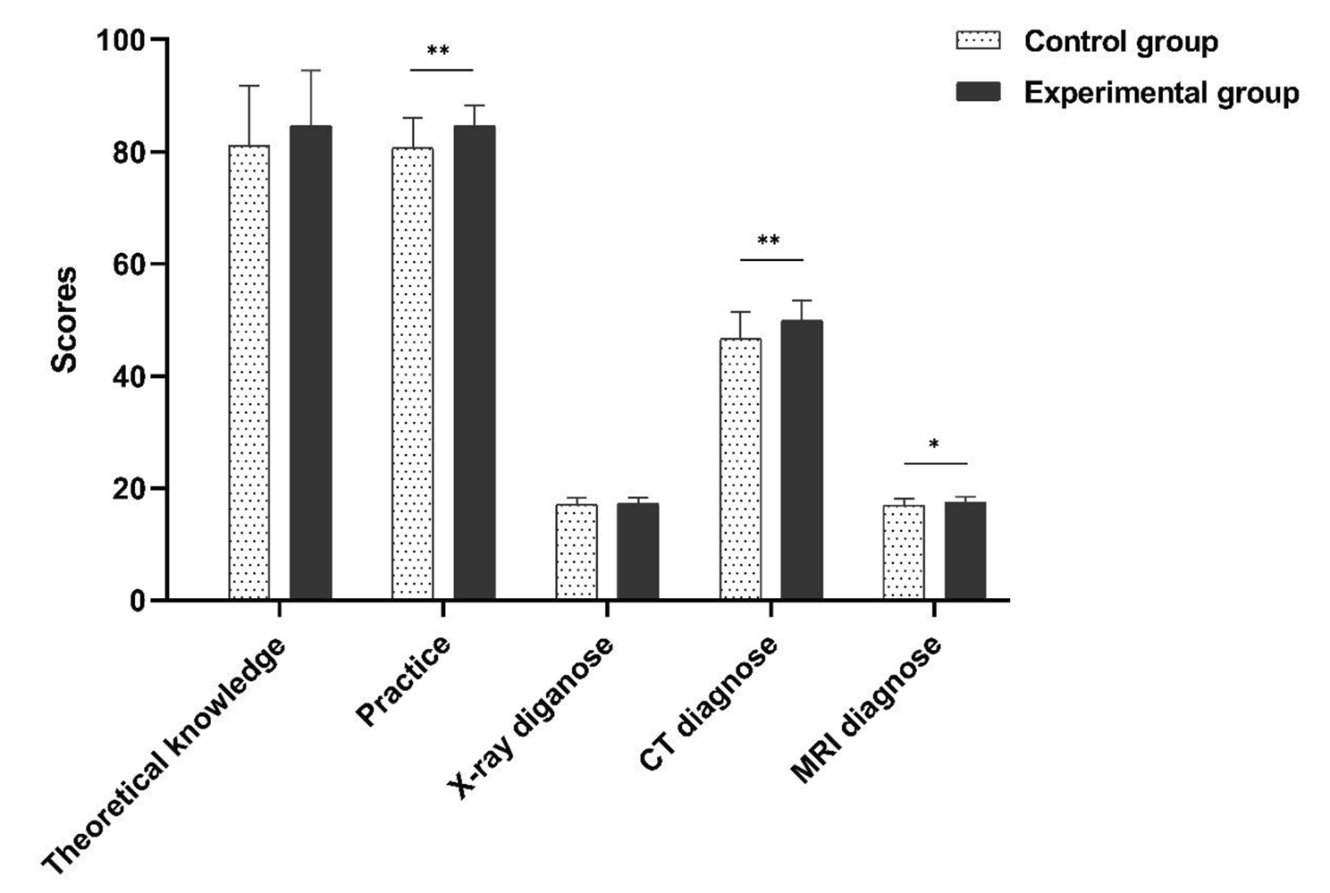
Comparison of exam scores. *Indicates < 0.05; **Indicates < 0.01

Comparison of exam results: The scores of the theoretical exam were not significantly different between groups (*P* > 0.05). The experimental group showed significantly higher total skill score than that in control group (*P* < 0.05). On this basis, the scores of X-ray diagnosis, CT diagnosis and MR diagnosis were compared between groups. Except the X-ray diagnosis, the scores of CT diagnosis and MRI diagnosis in the experimental group were both significantly increased than that in the control group (*P* < 0.05) (Table 1; Fig. 5).

**Table 1:** Comparison of exam results (Mean ± SD)

Exam item	Experiment group	Control group	Z	*P*
Theoretical exam	84.63 ± 9.82	81.09 ± 10.65	1.891	0.059
Total skill	84.51 ± 3.81^a^	80.67 ± 5.43 ^a, b^	3.252	0.001
X-ray diagnosis	17.17 ± 1.14	17.13 ± 1.08	0.097	0.923
CT diagnosis	49.93 ± 3.59	46.60 ± 4.89 ^a^	3.216	0.001
MRI diagnosis	17.41 ± 1.00	16.93 ± 1.14	2.133	0.033

Note: a, normal distribution was confirmed by Shapiro-Wilk test; b, equal variance was not assumed; the data was represented as mean ± standard deviation (SD), and Mann-Whitney U test was conducted for data analysis

**Fig. 6:**
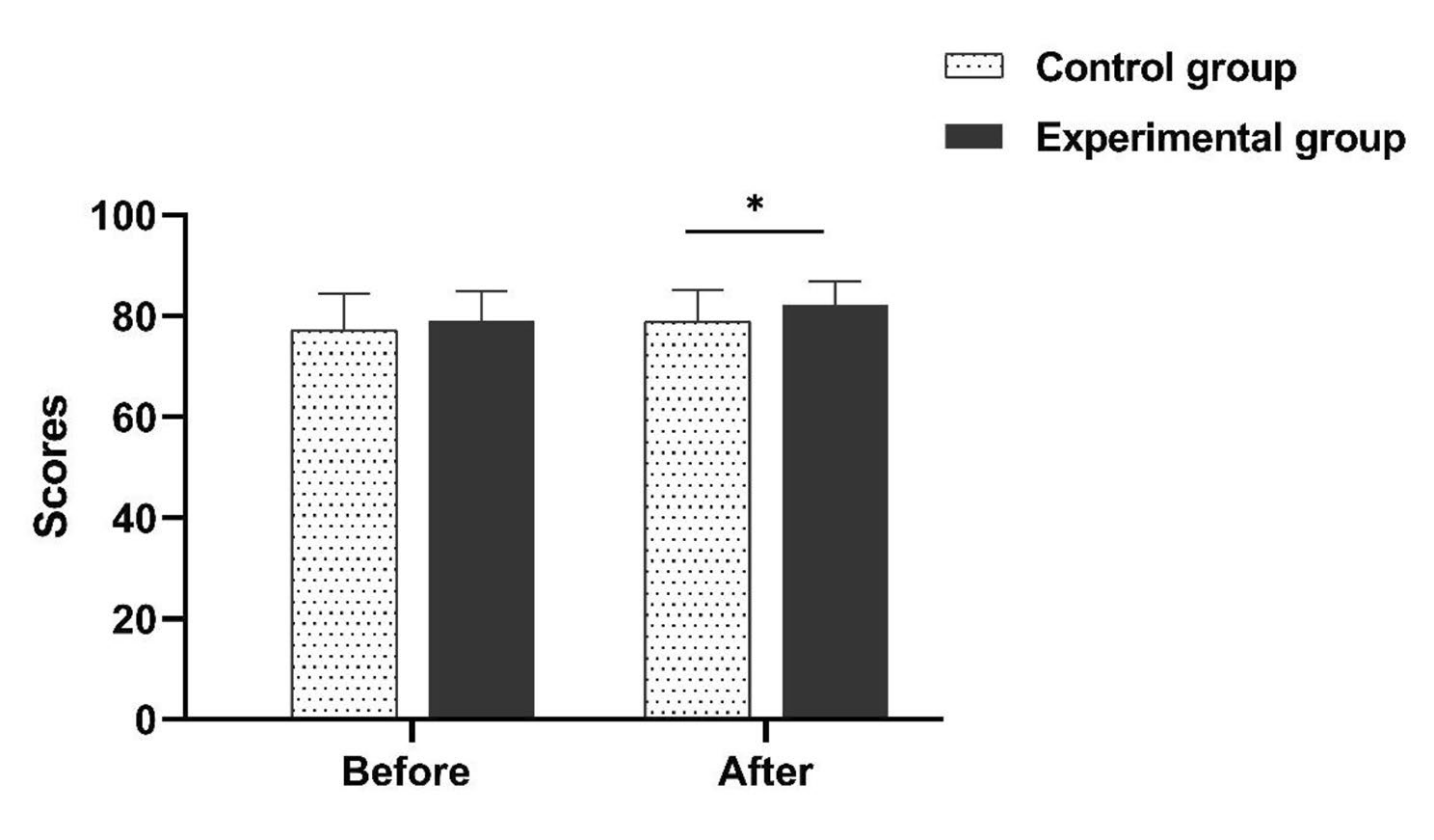
Comparison of academic self-efficacy before and after teaching implementation. *Indicates < 0.05

**Fig. 7:**
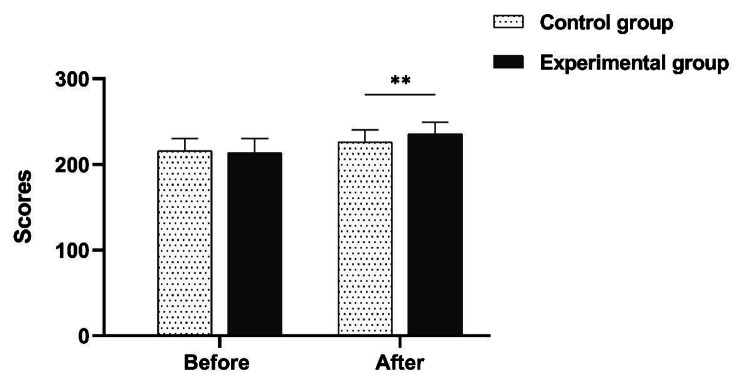
Comparison of self-directed learning abilities before and after teaching implementation. **Indicates < 0.01

In addition, the academic self-efficacy and self-directed learning ability was deeply discussed. The result showed that neither the academic self-efficacy nor self-directed learning ability was significantly different between the experimental group and control group before teaching initiated (*P* > 0.05). While after the teaching, the scores of academic self-efficacy and self-directed learning ability of the experimental group were both showed significantly increasing compared to the control group *(P* < 0.05) (Table 2; Figs. 6 and 7).

**Table 2:** Comparison of academic self-efficacy and self-directed learning ability (Mean ± SD)

Teaching evaluation	Evaluation item	Experiment group	Control group	Z	*P*
Before teaching	Academic self-efficacy	79.12 ± 5.83 ^a^	77.23 ± 7.28	1.161	0.246
	Self-directed learning ability	213.61 ± 16.72	216.19 ± 14.11	0.703	0.482
After teaching	Academic self-efficacy	82.17 ± 4.67	78.93 ± 6.29 ^a^	2.529	0.011
	Self-directed learning ability	235.56 ± 13.50	226.35 ± 13.90	3.349	0.001

Note: a, normal distribution was confirmed by Shapiro-Wilk test; the data was represented as mean ± standard deviation (SD), Mann-Whitney U test was conducted for data analysis

## Discussion

In this study, AISD software based on VDR was applied to practical teaching of imaging to help students better learn the course of medical imaging. Our research findings indicate that after implementation, students significantly improve their skill scores, enhance their academic self-efficacy, and enhance their self-learning abilities.

Kunpeng Zhang et al. applied AI software based on 3D reconstruction technology in aortic valve implantation surgery, which resulted in model imaging results closer to real images and more accurate preoperative predictions [14]. It can be seen that good results in clinical diagnosis and treatment have achieved by this software. During the practical teaching of medical imaging, AISD software based on VDR was introduced to solve the problems. First, the image data of typical clinical cases were introduced to form 3D images corresponding to the 2D images, thus forming structural adjacent relationships. Moreover, the students can revolve and magnify the images on computers. Our results showed the students in the test group had significantly higher academic self-efficacy than the control group, and clearer 3D images can better display the imaging features and better stimulate the students’ passion for learning. For instance, in the course of head and neck vasculopathy, cross-sectional 2D images of blood vessels can hardly make students think of stereoscopic structures of blood vessels [15]. The imaging features of intracranial aneurysm can be hardly understood, so when the students manually constructed 3D blood vessel images on the software, they can more profoundly understand the diseases. Additionally, AI can annotate the lesion positions on images, and improves the learning achievements in comparison with manual annotations. Khoynaroud et al. compared the flipped classroom teaching pattern and the traditional teaching, and found the students had better achievements in the flipped classroom than in the traditional classes [16]. Moreover, they used videos, books and case data before the classes in order to improve the students’ achievements. They introduced a new teaching pattern to improve students’ achievements, which is the same design clue of our study.

At present, VDR has been used in the medical field for imaging diagnosis, human body structure simulation, formulation of surgical plans, and development of image-guided surgery. Meanwhile, VDR technique should also be rapidly applied in the field of medical education [17, 18]. The AISD software based on VDR was built on basis of the DICOM images in PACS. Transaxial images shall be displayed as coronal and sagittal images, so as to construct visual VDR 3D images. The images at three directions can also be superimposed on VDR images, which largely narrowed down the difference between 2D images and the actual anatomy and solved the problems of image abstraction, thus facilitating the understanding and memorization of students. Yammine and Violato’s research indicated that more realistic spatial anatomy knowledge can be obtained through 3D software compared to traditional teaching methods [19]. The 3D images of tissues and organs (e.g. brain, heart, trachea, liver, spleen, gastrointestinal tract, skeleton, blood vessels) can be achieved through the hiding, subtraction, and hydrography techniques on the software. These VDR techniques all significantly outperform the traditional teaching. Like this study, Wang et al. found the 3D printed tooth model largely helped students understand the anatomic structure of teeth and improved their operating skills, increasing the achievement by 4.3 scores [20]. The experiment group outperformed the control group in terms of image reading skills, and the average score increased by 3.84, but the theoretical scores were not significantly different between groups, which are consistent with the findings of Alzahrani et al. [21]. The new teaching methods improve the students’ skills scores, but do not largely improve their theoretical knowledge scores. The reason may be that theoretical exams focus on the memorization of basic knowledge, but do not test clinical practice skills. The computer-based 3D visualization technology is far more operable than the 3D printing technique, which is because the materials of 3D printing models are much more expensive [22]. In addition, many studies had reported significant effects of virtual reality(VR) in education [23–26]. However, due to the large number of medical students in China, more VR devices were needed, including touch screens, joysticks, VR gloves, headphones, stereoscopic glasses, and so on, which require a significant amount of capital investment. Currently, we do not have sufficient funds to provide enough VR teaching equipments for every medical student. Compared with VR technology, VDR can also provide students with three-dimensional stereoscopic images for learning in our imaging practice teaching and research process. Interestingly, the achievement of X-ray diagnosis was not significantly improved, which may be because X-ray images are 3D and superimposed images and thus do not much improve the scores.

VR has developed based on scientific technologies such as computers, multimedia, human-computer interaction, and sensing. By constructing a virtual surgical plan through VR, trainers shortened the training time, and participants believed that VR was more intuitive [27]. This type of VR is different from the VDR involved in radiology. VDR is displayed as a three-dimensional structural diagram on a computer screen, or more accurately, as a three-dimensional display on a computer desktop. It does not require connecting more external devices such as touch screens, joysticks, stereo glasses, etc. VDR is commonly used by radiologists, such as rib 3D imaging and head and neck artery 3D imaging. Lesions can be more clearly displayed by VDR. Lennart et al.‘s paper discussed the accuracy and precision of VR and desktop screen display in mandibular segmentation, and found that VR is superior to desktop screen display [28]. Research has shown that VR has significant advantages in the application of medical education [29]. At the same time, research on VR mainly focuses on surgical virtual world simulators, virtual environments, and 3D anatomical models. As far as the three-dimensional anatomical model is concerned, it is developed from radiographic images. VDR has become a part of VR in three-dimensional anatomical models. In this study, we adopted a teaching method that does not require additional external devices and can display three-dimensional stereoscopic images using only a computer screen. Users can freely rotate images. The scores of student skills exams have significantly improved, which effectively helps to improve the quality of teaching practical skills. The three-dimensional display of lesions often provides students with a good perspective [30]. In a classroom led by a teacher, not all student positions are the best observation points. However, the truly optimal perspective can enhance learning effectiveness. This may make VDR the most powerful tool. In this study, participants were provided with VDR exercises to enhance their self-efficacy and multiple spatial exercises. This may help students better grasp the anatomical structure and positional relationships and increase their confidence in achieving learning goals. Chang et al. pointed out in their study on the pre care planning and decision-making of medical professionals in the VR teaching module that using the VR module can make medical staff more confident in implementing pre decisions [31]. Our research further supports the application of VDR in practical teaching of imaging, which can help students better learn the course of medical imaging and improve their academic self-efficacy.

Xiaoqin Zhang et al. [32] reported that an elective course was offered using 3D reconstruction technology combined with 3D printing. The average skill score of 30 students increased to 89.2 ± 3.4 points, and 90% of the students in the survey believed that medical image post-processing helped to gain a deeper understanding of anatomical structures and spatial relationships, which was similar to our research results. However, it is worth noting that their research did not use AI for auxiliary learning, and some students face complex imaging images, making it difficult to find the specific location of lesions and imaging diagnostic ideas. During imaging diagnosis, “different diseases with mimicking images” (diagnosis of different disease from the same image) and “the same disease with different images” (the same disease showing different imaging manifestations) shall be judged according to rich clinical experience [33]. However, the limitations in the learning hours and faculty [34] affected the students’ practical imaging reading ability. So far, AI has become a new direction of various industries, and more studies on the clinical application of diagnostic imaging AI have emerged [35, 36]. With the continuous development of medical image intelligent analysis technology, AI is playing an increasingly important role in auxiliary medicine [37]. However, there is little research on the application of AI into the teaching of imaging. The text annotations formed after AI analysis of images can be compared with the students’ diagnostic results, which modify their clinical thinking at any time, improve their ability to solve clinical problems, and relieve the teacher’s workload. The self-directed learning scale was introduced to evaluate the self-directed learning ability of the nursing students [38]. Evaluation with the scale showed that the learning ability of the test group was significantly higher than that of the control group. This was probably because the students’ problems can be corrected in time by AI, which was faster than waiting for an answer from the teacher. Such cheerful teaching experience further improves the students’ initiative for learning, which is consistent with the findings of Baid et al. [39].

Shortcomings of this study: Firstly, this article conducted a comparative study of students in 2016 and 2017, which is a retrospective study rather than a randomized controlled trial. We did not conduct a Mental Rotation Test (MRT) on students. In future research, MRT will be conducted on students before project implementation to achieve more accurate evaluations. Secondly, due to the small sample size, there may be selective bias. Subsequently, AISD soft will be used for all students who come to the hospital for internships to expand the sample size.

## Conclusions

This study found that using AISD software based on VDR technique for teaching medical imaging practical courses can improve the shortcomings of traditional teaching, enhance students’ image reading skills, help students build three-dimensional thinking, enhance self-efficacy and self-learning ability.

### Electronic supplementary material

Below is the link to the electronic supplementary material.

Supplementary Material 1

## Data Availability

Correspondence and requests for materials should be addressed to the corresponding author D.W.

## Abbreviations

AI: Artificial intelligence
CT: Computerized tomography
DICOM: Digital Imaging and Communications in Medicine
MR: Magnetic resonance
PACS: Picture Archiving and Communication System
VDR: Volume Data Reconstruction
VR: Virtual Reality

## References

[1] Yue JY, Chen J, Dou WG, Liang CH, Wu QW, Ma YY, Zhu ZP, Li MX, Hu YL (2018). Using integrated problem- and lecture-based learning teaching modes for imaging diagnosis education. BMC Med Educ.

[2] O’Keeffe GW, Davy S, Barry DS. Radiologist’s views on anatomical knowledge amongst junior doctors and the teaching of anatomy in medical curricula. Ann Anat. 2019;223:70–76. 10.1016/j.aanat.2019.01.011. Epub 2019 Feb 4. PMID: 30731200. 10.1016/j.aanat.2019.01.011 30731200

[3] Chen Y, Zheng K, Ye S (2019). Constructing an experiential education model in undergraduate radiology education by the utilization of the picture archiving and communication system (PACS). BMC Med Educ.

[4] Huang HK (2014). Medical imaging, PACS, and imaging informatics: retrospective. Radiol Phys Technol.

[5] Pujol S, Baldwin M, Nassiri J (2016). Using 3D modeling techniques to enhance teaching of difficult anatomical concepts. Acad Radiol.

[6] Mello-Thoms C (2023). Teaching Artificial Intelligence literacy: a challenge in the education of Radiology residents. Acad Radiol.

[7] Stefan P, Pfandler M, Lazarovici M (2020). Three-dimensional-printed computed tomography-based bone models for spine surgery simulation. Simul Healthc.

[8] Jiang F, Jiang Y, Zhi H, Dong Y, Li H, Ma S, Wang Y, Dong Q, Shen H, Wang Y (2017). Artificial intelligence in healthecare: past, present and future. Strole Vasc Neurol.

[9] Syed AB, Zoga AC (2018). Artificial intelligence in radiology: current technology and future directions. Seminars Musculskeletal Radiol.

[10] Yu Ito A, Miyoshi et al. An artificial intelligenceassisted diagnostic system improves the accuracy of image diagnosis of uterine cervical lesions. Mol Clin Oncol. 2022;16:27. 10.3892/mco.2021.2460 PMC8719259 34987798

[11] van Ginneken B, Schaefer-Prokop CM, Prokop M (2011). Computer-aided diagnosis: how to move from the laboratory to the clinic. Radiology.

[12] Duong MT, Rauschecker AM, Rudie JD (2019). Artificial intelligence for precision education in radiology. Br J Radiol.

[13] Wu H, Li S, Zheng J, Guo J (2020). Medical students’ motivation and academic performance: the mediating roles of self-efficacy and learning engagement. Med Educ Online.

[14] Zhang K, Gao Y, Lv J, Li J, Liu J (2022). Artificial Intelligence-based spiral CT 3D Reconstruction in Transcatheter aortic valve implantation. Comput Math Methods Med.

[15] Jacquesson T, Simon E, Dauleac C, Margueron L, Robinson P, Mertens P (2020). Stereoscopic three-dimensional visualization: interest for neuroanatomy teaching in medical school. Surg Radiol Anat.

[16] Khoynaroud AA, Akbarzadeh A, Ghojazadeh M, Ghaffarifar S (2020). Assessment of the effect of application of an educational wiki in flipped classroom on students’ achievement and satisfaction. BMC Med Educ.

[17] Azer SA, Azer S. 3D anatomy models and impact on learning: a review of the quality of the literature. Health Prof Educ. 2016;2(2):80–98. 10.1016/j.hpe.2016.05.002

[18] Brenton H, Hernandez J, Bello F, Strutton P, Purkayastha S, Firth T et al. Using multimedia and web3D to enhance anatomy teaching. Comput Educ. 2007;49(1):32–53. 10.1016/j.compedu.2005.06.005

[19] Yammine K, Violato C (2015). A meta-analysis of the educational effectiveness of three-dimensional visualization technologies in teaching anatomy. Anat Sci Educ.

[20] Wang H, Xu H, Zhang J, Yu S, Wang M, Qiu J, Zhang M (2020). The effect of 3D-printed plastic teeth on scores in a tooth morphology course in a Chinese university. BMC Med Educ.

[21] Alzahrani AA, Alhassan EM, Attia MA (2019). Enhancing dental carving skills of preclinical dental hygiene students using online dental anatomy resources. Open Dent J.

[22] Asif A, Lee E, Caputo M, Biglino G, Shearn AIU (2021). Role of 3D printing technology in paediatric teaching and training: a systematic review. BMJ Paediatr Open.

[23] Maresky HS, Oikonomou A, Ali I, Ditkofsky N, Pakkal M, Ballyk B (2019). Virtual reality and cardiac anatomy: exploring immersive three-dimensional cardiac imaging, a pilot study in undergraduate medical anatomy education. Clin Anat.

[24] Alsufyani N, Alnamlah S, Mutaieb S (2023). Virtual reality simulation of panoramic radiographic anatomy for dental students. J Dent Educ.

[25] Peters P, Lemos M, Bönsch A, Ooms M, Ulbrich M, Rashad A, Krause F, Lipprandt M, Kuhlen TW, Röhrig R, Hölzle F, Puladi B (2023). Effect of head-mounted displays on students’ acquisition of surgical suturing techniques compared to an e-learning and tutor-led course: a randomized controlled trial. Int J Surg.

[26] Kato K, Kon D, Ito T, Ichikawa S, Ueda K, Kuroda Y (2022). Radiography education with VR using head mounted display: proficiency evaluation by rubric method. BMC Med Educ.

[27] Ulbrich M, Van den Bosch V, Bönsch A (2023). Advantages of a training course for Surgical Planning in virtual reality for oral and maxillofacial surgery: crossover study. JMIR Serious Games.

[28] Gruber LJ, Egger J, Bonsch A et al. Accuracy and precision of mandible segmentation and its clinical implications: virtual reality, desktop screen and artificial intelligence. Expert Syst Appl. 2024;239:122275. 10.1016/j.eswa.2023.122275

[29] Jiang H, Vimalesvaran S, Wang JK, Lim KB, Mogali SR, Car LT (2022). Virtual reality in medical students’ education: scoping review. JMIR Med Educ.

[30] Peters P, Lemos M, Bönsch A (2023). Effect of head-mounted displays on students’ acquisition of surgical suturing techniques compared to an e-learning and tutor-led course: a randomized controlled trial. Int J Surg.

[31] Chang YK, Wu YK, Liu TH (2024). The effectiveness of a virtual reality teaching module on advance care planning and advance decision for medical professionals. BMC Med Educ.

[32] Zhang X, Xu Z, Tan L (2019). Application of three-dimensional reconstruction and printing as an elective course for undergraduate medical students: an exploratory trial. Surg Radiol Anat.

[33] Elsayes KM, Khan ZA, Kamel S, Rohren S, Patel P, Ghannam S, Baqai F, Aly MA, Gopal A, Reiter AM (2021). Multidisciplinary Approach in Teaching Diagnostic Radiology to Medical students: the development, implementation, and evaluation of a virtual Educational Model. J Am Coll Radiol.

[34] Kamel S, Wang MX, Ghannam S, Gopal A, Baqai F, Rohren S, Patel P, Khan Z, Aly M, Reiter AM, Zook S, Udayakumar N, Kumaraval M, Kamaya A, Jambhekar K, Elsamaloty H, Gaballah A, Stein L, Abdelsalam M, Chernyak V, Elsayes KM. Acing the fundamentals of radiology: an online series for medical students and interns. J Comput Assist Tomogr. 2022;46(4):614–20. 10.1097/RCT.0000000000001306. Epub 2022 Apr 8. PMID: 35405708. 10.1097/RCT.0000000000001306 35405708

[35] Hosny A, Parmar C, Quackenbush J, Schwartz LH, Aerts HJWL (2018). Artificial intelligence in radiology. Nat Rev Cancer.

[36] Hardy M, Harvey H (2020). Artificial intelligence in diagnostic imaging: impact on the radiography profession. Br J Radiol.

[37] Gemescu IN, Thierfelder KM, Rehnitz C, Weber MA (2019). Imaging features of bone tumors: conventional radiographs and MR Imaging correlation. Magn Reson Imaging Clin N Am.

[38] Cadorin L, Bressan V, Palese A (2017). Instruments evaluating the self-directed learning abilities among nursing students and nurses: a systematic review of psychometric properties. BMC Med Educ.

[39] Baid H, Lambert N (2010). Enjoyable learning: the role of humour, games, and fun activities in nursing and midwifery education. Nurse Educ Today.

